# Shift of microRNA profile upon glioma cell migration using patient-derived spheroids and serum-free conditions

**DOI:** 10.1007/s11060-016-2356-x

**Published:** 2017-01-13

**Authors:** Sune Munthe, Bo Halle, Henning B. Boldt, Helle Christiansen, Steffen Schmidt, Vivek Kaimal, Jessica Xu, Sonya Zabludoff, Jan Mollenhauer, Frantz R. Poulsen, Bjarne W. Kristensen

**Affiliations:** 1 Department of Pathology, Odense University Hospital, Winsløwparken 15, 3rd floor, 5000 Odense C, Denmark; 2 Institute of Clinical Research, University of Southern Denmark, Winsløwparken 19, 5000 Odense C, Denmark; 3 Department of Neurosurgery, Odense University Hospital, Sdr. Boulevard 29, 5000 Odense C, Denmark; 4 Department of Nuclear Medicine, Odense University Hospital, Sdr. Boulevard 29, 5000 Odense C, Denmark; 5 Faculty of Health Sciences, Lundbeckfonden Center of Excellence NanoCAN and Molecular Oncology, Institute of Molecular Medicine, University of Southern Denmark, Winsløwparken 25, 5000 Odense C, Denmark; 6 Regulus Therapeutics, San Diego, CA USA

**Keywords:** Glioblastoma, Migration, Serum-free, MicroRNA, Target

## Abstract

Glioblastoma multiforme (GBM) is the most frequent malignant primary brain tumor. A major reason for the overall median survival being only 14.6 months is migrating tumor cells left behind after surgery. Another major reason is tumor cells having a so-called cancer stem cell phenotype being therefore resistant towards traditional chemo- and radiotherapy. A group of novel molecular targets are microRNAs (miRNAs). MiRNAs are small non-coding RNAs exerting post-transcriptional regulation of gene expression. The aim of this study was to identify differentially expressed miRNAs in migrating GBM cells using serum-free stem cell conditions. We used patient-derived GBM spheroid cultures for a novel serum-free migration assay. MiRNA expression of migrating tumor cells isolated at maximum migration speed was compared with corresponding spheroids using an OpenArray Real-Time PCR System. The miRNA profiling revealed 30 miRNAs to be differentially expressed. In total 13 miRNAs were upregulated and 17 downregulated in migrating cells compared to corresponding spheroids. The three most deregulated miRNAs, miR-1227 (up-regulated), miR-32 (down-regulated) and miR-222 (down-regulated), were experimentally overexpressed. A non-significantly increased migration rate was observed after miR-1227 overexpression. A significantly reduced migration rate was observed after miR-32 and miR-222 overexpression. In conclusion a shift in microRNA profile upon glioma cell migration was identified using an assay avoiding serum-induced migration. Both the miRNA profiling and the functional validation suggested that miR-1227 may be associated with increased migration and miR-32 and miR-222 with decreased migration. These miRNAs may represent potential novel targets in migrating glioma cells.

## Introduction

Glioblastoma multiforme (GBM) is the most common and malignant primary brain tumor in adults. Despite multi-modal treatment options including surgery, radiation and chemotherapy, patients with GBM have an overall poor outcome with a mean survival of 14.6 months [1] and a 5-year survival rate of 9.8% [2]. Gliomas are highly invasive and migrate into the normal brain parenchyma along vessels and white matter fiber-tracts [3]. Therefore, migrating tumor cells will always be left behind despite surgery. The underlying phenotype characterizing highly aggressive migrating tumor cells causing short survival and treatment failure is not yet fully understood. As cancer stem cells (CSCs) have been shown to be both radio- and chemoresistant [4, 5], this may explain part of the resistance of migrating glioma cells.

Mature microRNAs (miRNAs) are short single-stranded non-coding RNAs involved in post-transcriptional regulation of numerous different target messenger RNAs (mRNAs). The miRNAs are key regulators, reported to regulate up to 60% of all mammalian genes [6, 7]. In GBMs several over-expressed miRNAs have been identified [8, 9] and inhibition of some of these diminish cellular proliferation rates [10–12] and angiogenesis [12]. This suggests novel therapeutic possibilities in the treatment of GBM.

Using different migration assays, numerous groups have identified several miRNAs associated with migration or invasion [9–13]. However, all employed migration assays have been performed with the use of fetal calf serum in the culture medium. Because fetal calf serum influences the tumor cell phenotype [14–16] and may influence expression of miRNA involved in migration the use of a recently developed serum-free migration assay was preferred [17].

The aim of this study was to discover novel miRNAs associated with GBM tumor cell migration. Five GBM spheroid cultures with tumorigenic stem-like cells were used, three previously established in our group [18–20] and two established as a part of the present study. To isolate migrating cells from non-migrating GBM cells at optimal time-points with fast migration, we used a flat surface migration assay allowing easy monitoring and isolation of migrating GBM cells. We recently established such an assay using serum-free so-called stem cell medium in an attempt to allow migrating cells to preserve a potential cancer stem cell phenotype [17]. MiRNA profiling was performed on the migrating cells and corresponding spheroids to identify deregulated miRNAs. In addition, we functionally evaluated the effect of experimental over-expression of the three most deregulated miRNAs in relation to migration.

## Materials and methods

### Culturing of cells

**Fig. 1:**
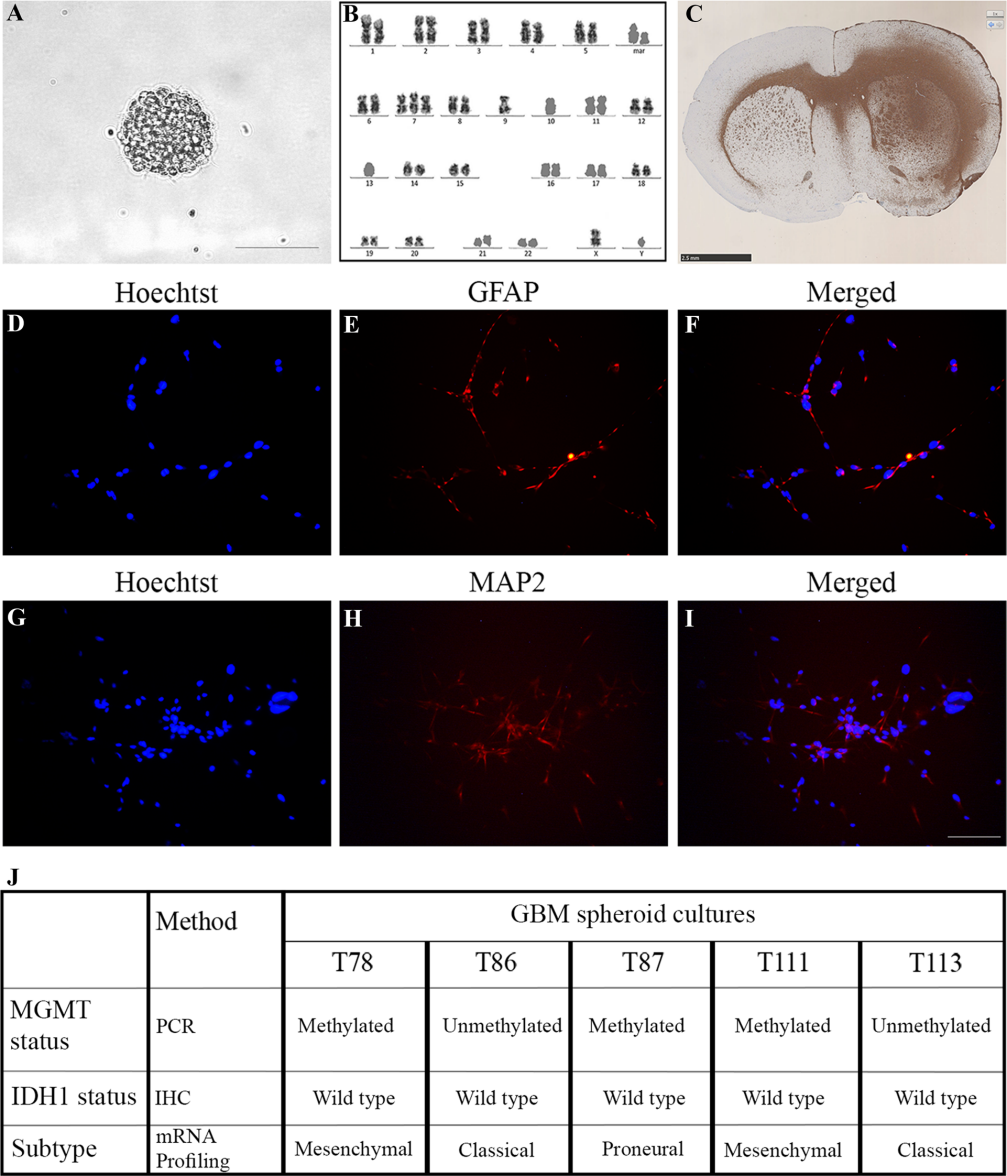
Characterization of patient-derived GBM spheroid cultures. The two GBM spheroid cultures established (T111 and T113) were cultured in serum-free medium as spheroids which upon trypzination to single cells developed new spheroids (**a**). The spheroid cultures were karyotyped and common characteristics were found such as gain of chromosome 7, found in both spheroid cultures, and loss of chromosome 10 (only T111) (**b**). Large tumors with highly migratory capacity upon orthotopic xenografting were formed (**c**). A differentiation assay was performed showing expression of the astrocytic marker GFAP (**d**–**f**) and the neuronal marker MAP2 (**g**–**i**). The MGMT status for both GBM spheroid cultures were performed with a pyrosequencing assay. T111 was found to be methylated and T113 to be unmethylated. Both were derived from IDH1 wild type tumors, representing primary GBMs. The molecular subtype based on mRNA profiling revealed T111 as mesenchymal and T113 as classical. An overview of MGMT, mIDH1 and subtype status of T111 and T113 and the three remaining spheroids cultures is given in (**j**). All illustrations are shown for T111. Scalebar in **a** 100 µm, **c** 2.5 mm and **d**–**i** 100 µm

Five different GBM spheroid cultures were utilized [18–20]. Three were previously established in our group (T78, T86, T87) [18–20] and two were established as a part of the present study (T111, T113). These cultures have the ability to form new spheroids at clonal density, have a karyotype typical of GBMs and the ability to differentiate into cells expressing neuronal, astrocytic and oligodendrocytic markers upon culturing in fetal calf serum medium, as well as the ability to form highly invasive tumors upon orthotopic xenografting (Fig. 1). The GBM spheroid cultures and recombinant U251 cells transfected with miRNA constructs were grown as free-floating spheroids in serum-free medium [18–20]. The serum-free medium contained Neurobasal A (Life Technologies), 1% B27 without vitamin A (Life Technologies), 0.5% N2 (Life Technologies), 1% Penicillin–streptomycin (Life Technologies), 1% Glutamine (Life Technologies), 25 ng/mL EGF(Epidermal Growth Factor) (Sigma Aldrich), 25 ng/mL bFGF (basic Fibroblast Growth Factor) (Trichem). All cultures were kept at 36 °C and 5% CO_2_. Permission to use tumor tissue was given by the Regional Scientific Ethical Committee (approval number S-VF-20040102). Tissue was obtained after informed consent.

The commercial cell line U251 was used for miRNA transfection, since in our experience the patient-derived cultures are difficult to transfect. These cultures are more patient-like but on the other hand also most likely polyclonal and more heterogeneic to work with.

### Migration assay

The migration assay used was established in a recently published parallel study [17], where all five GBM spheroid cultures were included and characterized with respect to migration distance and migration speed. This flat surface migration assay allowed monitoring of cell migration and easy isolation of migrating cells for further analysis. Each well, in a 12 well plate, was coated with a mixture of Geltrex (Gibco) and serum-free medium (1:49) and incubated at 36 °C and 5% CO_2_. The supernatant was removed the following day and a single spheroid was isolated and placed in each coated well. Afterwards one ml serum-free medium was added to each well and incubated at 36 °C and 5% CO_2_. For comparison of the commercial cell line U251 used for the transfection studies to the five GBM spheroid cultures, time-lapse microscopy was performed at 36 °C and 5% CO_2_, with pictures taken every hour for 5 days in all five GBM spheroid cultures including. From the time-lapse microscopy images we measured and calculated migration distance and speed using ImageJ, an open source program. Migrating cells and corresponding spheroids were isolated at the time point of highest migration speed in a new set of experiments. The non-migrating cells still shaped as a spheroid were removed under a microscope with a micropipette where after the residual migratory cells were isolated after 48 h of migration. Cells were then placed in Trizol (Life technologies) and vortexed to ensure lysis of cells. Finally they were frozen before miRNA profiling was done.

The migration assay was upon miRNA transfection performed in duplicate with a 12 well plate in each experiment. Doxycycline was used to activate the over-expression of the transfected miRNA.

### MicroRNA profiling

After thawing, chloroform at 20% volume was added to the cells to ensure proper lysis. Total RNA was purified from migrating spheroids (40 × 12 well plates) and corresponding free floating spheroids according to the manufactures protocol using the miRNeasy Mini Kit (Qiagen). Total RNA concentration was determined by Ribogreen [21] and samples were dried down in a speedy vac to suitable volume. A concentration of 30 ng/μl was used for Open Array Analysis. Four μl of RNA extract was used as sample input for miRNA profiling on the OpenArray® Real-Time PCR System (based on miRBase v. 14 database which detect more than 700 miRNAs) using the manufacturer’s instructions (Applied Biosystems, Life technologies, Carlsbad, CA). For cultures, where more RNA extract than 4 µl was available, the extra material was used for extra replicates. Briefly, Megaplex™ RT Primers, Human Pool A v.2.1 and Megaplex™ RT Primers, Human Pool B v.3.0 were used for the reverse transcription step. Megaplex™ PreAmp Primers, Human Pool A v.2.1 and Megaplex™ PreAmp Primers, Human Pool B v3.0 were used for the PreAmp step.

### Bioinformatic analysis on microRNA data

OpenArray® miRNA profiling data were processed and filtered using Perl and R scripts. Briefly, pre-filtering of data was performed to include only reliable Amp Scores (>1.15) and ΔCts in the range of 10–28. ΔCts above or below the range were considered unreliable, and hence “undetected”. Further analyses were done in R (http://www.R-project.org). Global mean normalization was used to normalize across samples. Paired differential expression analysis of miRNA expression between migrating cells and corresponding spheroids was done in the Bioconductor package *limma* for R (http://www.bioconductor.org). Benjamini and Hochberg’s method [22] to control the false discovery rate (FDR) was utilized to adjust the p-values for multiple testing.

### Cloning of the synthetic miRNA constructs

The synthetic miRNA constructs from the top three upregulated and top three downregulated miRNAs followed the Life-technologies algorithm for miRNA embedded short hairpin RNA (shRNA) expression. It uses the regulatory sequences of the murine miR-155. The design of the sequences was done according to miRBase 21 annotation as described by Hou et al. [23] as well as chemically synthesized as approximate 65-mer DNA oligos (MWG-Biotech, Eurofins). Top strand and bottom strand were annealed to form a double stranded DNA fragment with sticky ends and ligated into the linearized pcDNA™ 6.2-GW/miR vector (Invitrogen) with compatible overhangs. The EmGFP-miR-neg Control construct (Invitrogen) was used as negative control. The integrity of the constructs were confirmed by sequencing with M13 primers and transferred via Gateway cloning into the pDest1a expression vector which drives the expression of the miRNA constructs under the control of a tetracycline inducible cytomegalovirus (CMV) promoter [24].

#### Transfection of cells with plasmid DNA

Cells were seeded at a density of 6.5 × 10^4^ cells per well in 6-well plates. The next day medium was replaced with fresh growth medium (DMEM medium supplemented with 10% fetal calf serum, 2mM l-Glutamine and 100 µ/mL Pen-Strep) and the expression plasmids co-transfected with pOG44 plasmid using GeneJammer transfection reagent. After 48 h of incubation, the medium was changed with selection medium containing 250 µg/ml hygromycin. The medium was exchanged 2–3 times per week and non-green fluorescent colonies expanded as individual recombinants. Thereafter the medium was changed to serum-free medium. Cells were cultured as free floating spheroids and passaged 5 times until the migration assay was performed.

Since the recombinant cell lines contain the respective miRNA constructs under the control of a tetracycline inducible promoter, 50 ng/ml Doxycycline (Life Technologies™) was added to the medium 48 h before experiments were conducted to activate the expression of the miRNA constructs. MiRNA transfection to U251 was performed twice.

The migration distance was measured for 48 h after start of the migration assay since the highest migration speeds were seen within the first 48 h.

### Real-time PCR

Total RNA extraction was performed with RNeasy Mini Kit (Qiagen) including On-column DNase Digestion with RNase-Free DNase set (Qiagen). Prior to processing, sample RNA concentration was measured using a Nanodrop™ 2000 (Thermo Scientific). Changes in expression of miR-32 ± Doxycycline, miR-222 ± Doxycycline and miR-1227 ± Doxycycline were evaluated using TaqMan microRNA RT kit and Megaplex RT Primers (Life Technologies™) and miRNAs were pre-amplified using TaqManPreAmp Master mix and PreAmp Primer Pools, Human Pools Set v3.0 (Life Technologies™); reverse-transcription and pre-amplification were performed with the thermocycler GeneAmp PCR system 9700 (Life Technologies™) using the protocol for low sample input from the manufacturer (http://www.tools.lifetechnologies.com). MiRNA quantification was performed by Real-Time PCR using the standard protocol of TaqManOpenArray Human miRNA Panel and the QuantStudioTM 12 K Flex Real-Time PCR System (Life Technologies™). Hsa-miR-16 was used for normalization. All reactions were performed in duplicates. Data was analyzed in Biogazelle qBase + ver. 3.0. Exclusion criteria were the following: Cq confidence lower than 0.8, Ct standard deviation in replicate group higher than 0.5, no amplification (amplification algorithm results lower than 0.1), noise spikes (spikes algorithm results higher than 1) and Ct higher than 32.

### Statistical analysis

Comparisons of miRNA profiling were performed using Benjamini and Hochberg’s analysis. Comparison of miRNA expression and migration distance were performed with student’s t-test. Statistical significance was considered at p < 5%.

## Results

**Fig. 2:**
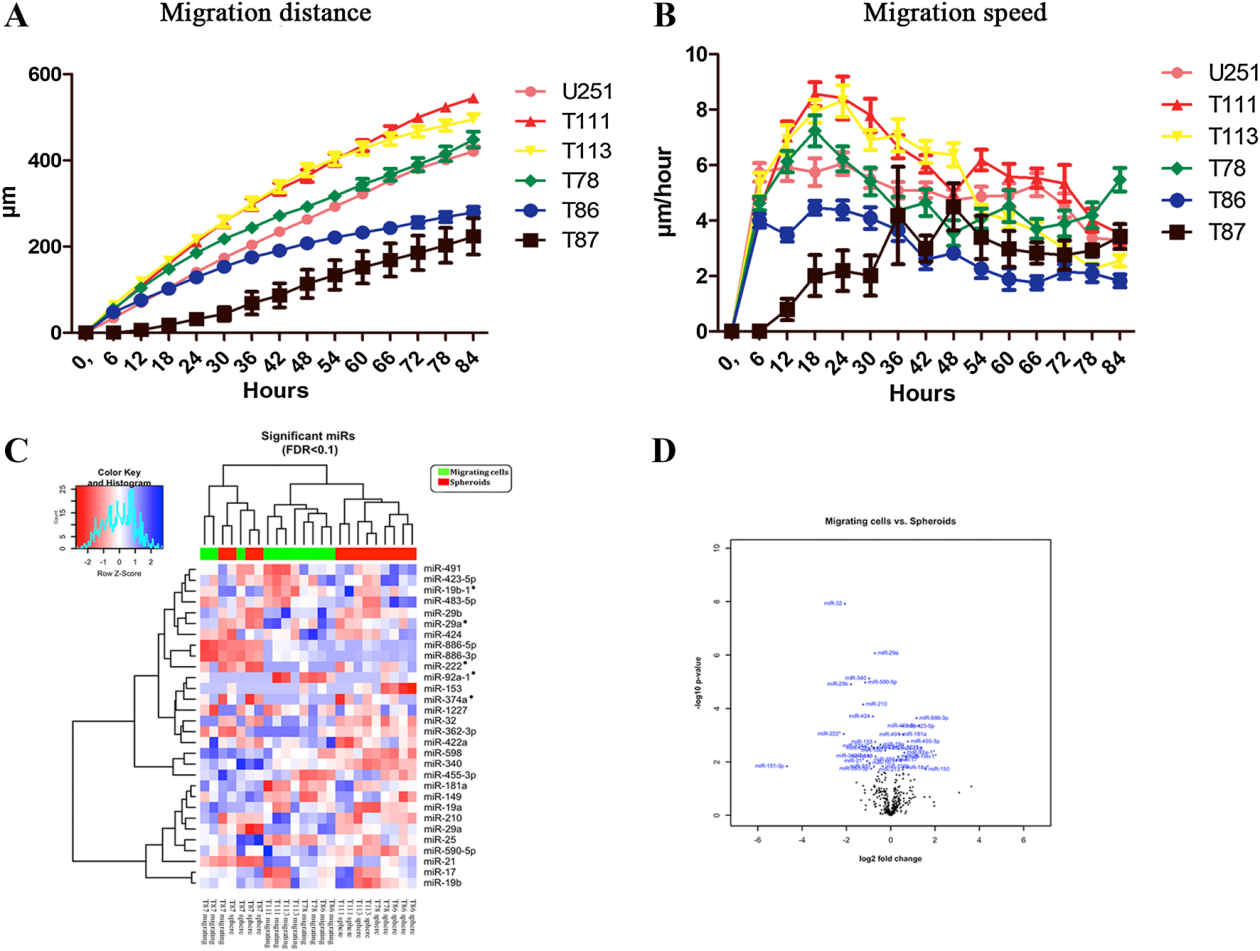
Migration time-lapse microscopy showing results for the five GBM spheroid cultures and the U251 cell line. Migration distance was measured for all spheroid cultures and U251 over five days (**a**). Migration speed was calculated for each spheroid culture and the U251 cell line (**b**). Migration data for the GBM spheroid cultures were obtained in a separate study and included here for comparison [17]. The miRNA profiling of isolated migrating cells and corresponding spheroids revealed a clustering of significantly deregulated microRNAs in both migrating and corresponding spheroids (**c**). The volcano plot illustrates the microRNA deregulation pattern in migrating cells (**d**). Data are shown as means ± SEM, n = 22 for each spheroid culture or cell line

The original U251 cell line was capable of migration reaching a distance and speed similar to the five GBM spheroid cultures earlier characterized [17]. The serum-free migration assay combined with time-lapse microscopy revealed continued migration for a duration of 5 days (Fig. 2a). A maximum migration speed was reached after 18 h for all cultures except the T87 GBM spheroid culture, which reached a maximum after 48 h (Fig. 2b). The time-point of highest migration-speed was interesting based on an assumption that miRNAs related to migration would be most deregulated when cells were migrating at maximum speed. The miRNA profiling of all five GBM spheroids and their corresponding migrating cells revealed 30 significantly deregulated miRNAs (Table 1). In total 13 miRNAs were found to be upregulated and 17 downregulated in the migrating cells compared to the levels found in the corresponding spheroids. Three miRNAs (miR-1227, miR-483-5p and miR-886-3p) were found to be more than twofold upregulated in the migrating cells. Three miRNAs (miR-32,miR-222 and miR-29b) were more than threefold downregulated in the migrating cells. The heatmap from the miRNA profiling revealed a separate clustering of migrating cells and corresponding spheroids. The heatmap was based on miRNAs being significantly deregulated after multiple testing (Fig. 2c). Interestingly the T87 GBM spheroid culture, the only of Proneural subtype—revealed a clustering of migrating cells with the corresponding spheroids (Fig. 2c). The volcano plot shows distribution of these miRNAs (Fig. 2d).

**Table 1:** MicroRNA profiling of non-migrating and correspondingly migrating GBM cells from five GBM spheroid cultures (T78, T86, T87, T111 and T113)

Upregulated			Downregulated		
miR name	FC	P value	miR Name	FC	P value
miR-1227	2.61	0.00295752	miR-19b	−1.19	0.00373486
miR-483-5p	2.44	0.00045556	miR-19a	−1.38	0.0022409
miR-886-3p	2.24	0.0002296	miR-21	−1.52	0.00301401
miR-455-3p	1.71	0.00172537	miR-598	−1.60	0.00625604
miR-19b-1*	1.62	0.00640601	miR-153	−1.62	0.00179931
miR-886-5p	1.61	0.00836716	miR-29a	−1.65	8.5462E-07
miR-92a-1*	1.53	0.00448481	miR-29a*	−1.69	0.00288156
miR-491	1.47	0.00095984	miR-422a	−1.73	0.0031213
miR-423-5p	1.45	0.00046269	miR-424	−1.75	0.00019871
miR-181a	1.32	0.00092756	miR-374a*	−1.82	0.00257081
miR-149	1.27	0.00330589	miR-340	−1.96	7.5167E-06
miR-25	1.26	0.0062177	miR-362-3p	−2.02	0.00598894
miR-17	1.19	0.00846355	miR-590-5p	−2.22	1.0611E-05
			miR-210	−2.36	7.0884E-05
			miR-29b	−3.46	1.2505E-05
			miR-32	−4.20	1.2091E-08
			miR-222*	−4.35	0.00090445

The fold changes represent the average of all five cultures and are listed for the 30 deregulated miRNAs identified

Transfection of the top three and bottom three deregulated miRNAs was successfully achieved for miR-1227, miR-32 and miR-222. Although transfections were performed twice a successful transfection of miR-483-5p and miR-29b could not be achieved. The miR-886-3p was at the time of transfection no longer determined as a miRNA according to the miRBase v. 21 (http://www.miRBase.org). Thus miR-886-3p was omitted from the study.

**Fig. 3:**
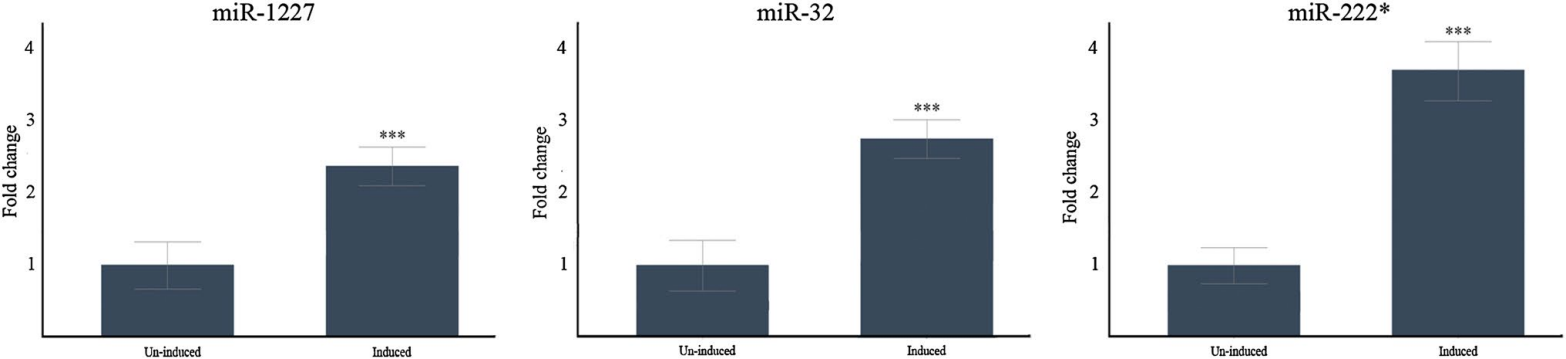
qPCR results on recombinant U251 cell lines with conditional expression of miR-32, miR-222 and miR-1227 constructs. Results obtained both with un-induced and (doxycycline) induced expression of the miRNAs. Endogenous miR-16 was used as reference. All miRNA constructs were significantly upregulated after induction with Doxycline. MiR-1227 was upregulated 2.4 fold, mir-32 2.8 fold and miR-222 3.78 fold. Data are shown as means ± SEM, n = 4, comparisons were made with student’s t-test. ***P < 0.001

When qPCR results were normalized to miR-16 there was an increased miR expression after induced overexpression of miR-1227 (2.4 fold) (Fig. 3a), miR-32 (2.6 fold) (Fig. 3b) and miR-222 (3.8 fold) (Fig. 3c).

**Fig. 4:**
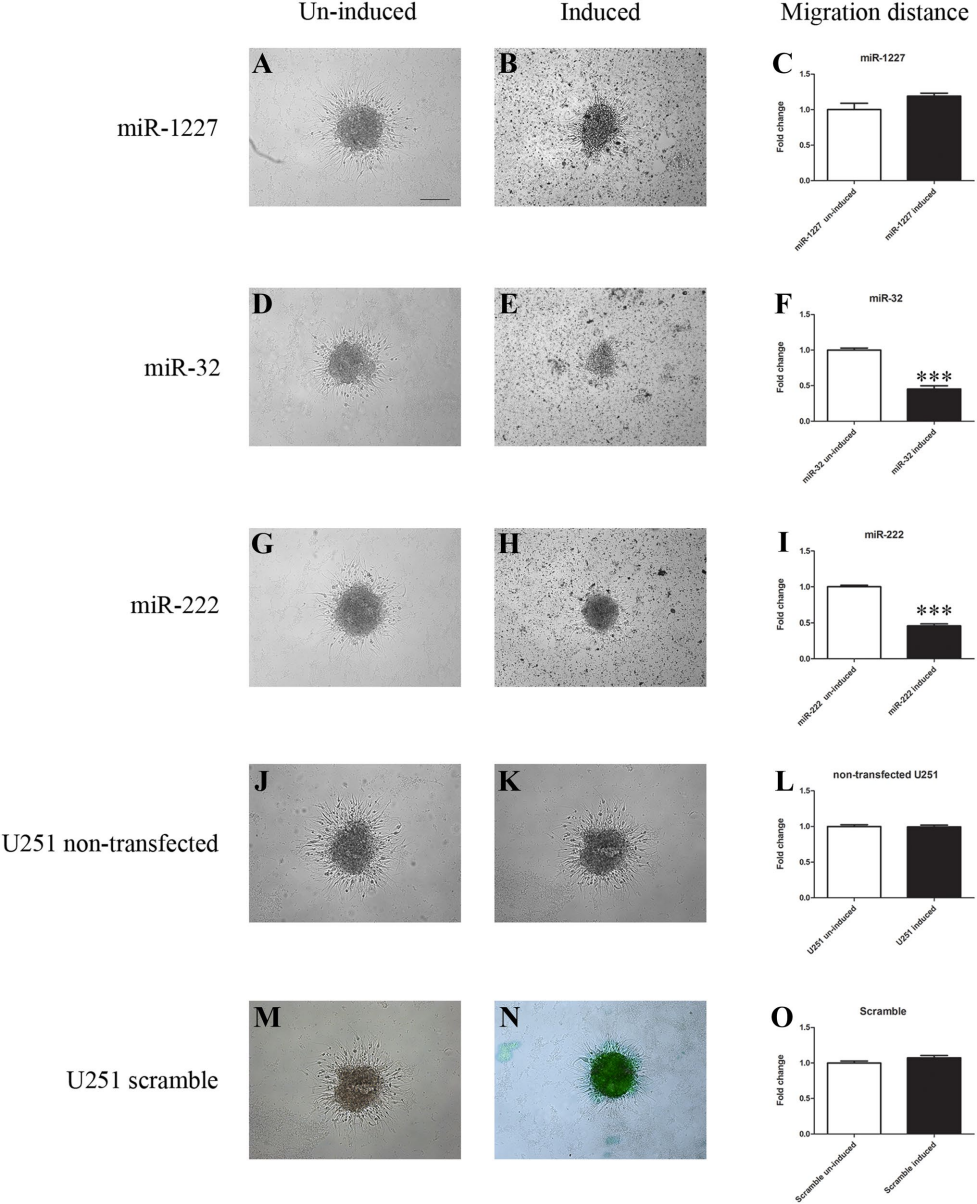
Migration assay over 48 h with four recombinant U251 cell lines (miR-1227, miR-32, miR-222, miR-control) and the original U251 cell line. MiRNA overexpression was induced with doxycycline. MiR-1227 revealed a non-significant increase in migration after overexpression. MiR-32 and miR-222 revealed a significantly impaired migration after overexpression. Both the recombinant U251 with the miR-control construct and the original U251 cell line showed no difference in migration upon addition of doxycycline. Data are shown as means ± SEM, n = 12, comparisons were made with student’s t-test. ***P < 0.001. Scale bar 100 µm

Upon induced overexpression of miR-1227 in U251 cells, a 1.2-fold increase in migration distance was measured after 48 h, although it was not statistical significant (Fig. 4a–c). Upon induced overexpression of miR-32, a 0.45-fold significant decrease in migration distance was measured (Fig. 4d–f). Upon induced overexpression of miR-222 (Fig. 4g–i) a 0.46-fold significant decrease in migration distance was measured. Both the original U251 cells and the recombinant cell line with inducible expression of the EmGFP-miR-neg control served as reference and revealed no difference in migration distance upon induction with Doxycycline (Fig. 4j–o).

## Discussion

The overall aim of this study was to identify deregulated potentially targetable miRNAs in migrating GBM cells. For this purpose, a newly established serum-free migration assay, presumably preserving the stem cell phenotype better than migration assays relying on serum, was used. We identified 30 miRNAs being deregulated in the migrating cells. MiR-1227 was identified as upregulated in the migrating cells and induced overexpression resulted in a non-significant increased migration distance after 48 h. MiR-32 and miR-222 were both downregulated in migrating GBM tumor cells compared to corresponding spheroids. Experimental upregulation of these two miRNAs significantly depressed migration.

We used patient-derived GBM spheroid cultures for miRNA profiling. The profiling was performed on migrating cells and corresponding spheroids in five different cultures, three previously established in our group and two established during this study. It strengthens the results that the cultures were patient-derived compared to results obtained by profiling commercial cell lines like U87MG [25]. Several groups including our group, have published that U87MG is unable to migrate in an orthotopic xenograft model [26], which is in line with what we find in our migration assay. Since we performed the miRNA profiling on cells maintained in a serum-free migration assay, the changes in miRNAs did not reflect a change due to fetal calf serum induced de-differentiation [27, 28]. However, although our assay may reflect the in vivo situation better by avoiding serum, our results need validation. Different serum-free in vitro migration systems exist, i.e. the spheroid-brain-slice culture migration model [29, 30]. However, the spheroid-brain-slice culture migration model requires combined culturing of a mouse or rat brain slice with GBM tumor cells. This makes it challenging to isolate migrating as well as non-migrating GBM tumor cells for analysis without contamination with brain slice derived rat or mice cells.

In this study miR-32 was identified as downregulated in the migrating GBM cells and experimental overexpression lead to decreased migration in vitro. No other studies have so far evaluated miR-32 expression in relation to migration in GBMs. Suh et al. reported that miR-32 overexpression inhibited proliferation and growth in GBMs [31]. In that study, a miR-32 containing construct was transfected into the commercial cell line U87MG and orthotopic in vivo experiments revealed a significantly reduced tumor volume as well as prolonged survival upon overexpression. MiR-32 was found to result in p53 accumulation by directly targeting Mdm2 and TSC1, which are negative regulators of p53 and the mTOR (mammalian target of rapamycin) pathway, respectively, leading to inhibition of cellular proliferation. These mechanisms may also be involved in the decreased migration observed in our study. However, the effects of miR-32 appear to be different in different cancers. In liver cancer miR-32 overexpression appeared to be associated with both increased proliferation and migration [32] and increased migration was also found upon overexpression in colorectal cancer [33].

MiR-222 has been reported to be upregulated in gliomas [34–36] and the expression increases with higher WHO grade [35]. Overexpression has been associated with increased migration whereas inhibition of miR-222 reduced migration [34, 35]. Different mRNA targets have been reported where Yang et al. and Zhang et al. found the mRNA target to be within the tissue inhibitor of metalloproteinases (TIMP)-family, (TIMP2 [34], TIMP3 [35]), whereas Quintaville et al. found the target to be the phosphate protein PTPµ [36]. All three studies used either scratch and/or transwell migration assays in the presence of fetal calf serum. In contrast, by using a serum-free migration assay, we found that the expression of miR-222 was downregulated in migrating cells compared to corresponding spheroids, and that overexpression of miR-222 led to diminished migration. Use of fetal calf serum, a multicomponent strong chemoattractant, is the most likely explanation for different results after overexpression of miR-222. In addition to this, the studies where fetal calf serum was used also used monolayer cultures, whereas we used spheroid cultures with three dimensional interactions between cells. Searching on miR-222 using miRConnect 2 [37], identifies among other mechanistically associated genes TIMPs and Insulin-like growth factor binding protein 6 (IGFBP6) with known functional roles in tumor cell migration and invasion. Expression of these and related genes may easily depend on culture medium and a two dimensional versus three dimensional culture setting.

Out of the three most deregulated miRNAs, we identified miR-1227 to be 2.8-fold overexpressed in the migrating cells. Upon overexpression of miR-1227, we observed a non-significant increase in migration. The lack of significance may be explained by a further increase in migration distance being unattainable, although a higher number of experiments might support the results. No comparable studies in GBMs identifying miR-1227 as overexpressed in the migrating cell compartment exist [38] and miR-1227 has not previously been identified to have an effect on migration in gliomas. Supporting a more general role of miR-1227 in different cancers high levels of miR-1227 have also been associated with increased migration in prostate cancer [39], where human prostatic epithelial RWPE-2-derived oncosomes containing miR-1227 seemed to enhance migration of cancer-associated fibroblasts.

The heterogeneity in gliomas has been displayed in several studies [40] and four subtypes have been described by Verhaak et al. [41]. The five different GBM spheroid cultures used in this study have been subtyped with two mesenchymal (T78, T111), two classical (T86, T113) and one proneural (T87) subtype identified [17]. In line with the genetic heterogeneity known to be present in gliomas [40], miRNA heterogeneity also exists [42, 43]. This partly explains the isolated miRNA clustering of the T87 spheroid culture data with both migrating and non-migrating. Apparently these two populations of T87 cells are more similar than different migrating groups or the different groups of spheroids. Therefore, future miRNA profiling, or any other profiling studies in GBMs, should be considered to also include GBM subtype in the analysis.

## Conclusion

In our novel serum-free migration assay-based study we discovered 30 different miRNAs to be deregulated in migrating cells compared to corresponding spheroids using five GBM spheroid cultures. Both the miRNA profiling and the functional validation suggested that MiR-1227 may be associated with increased migration and miR-32 and miR-222 with decreased migration of glioma cells. These miRNAs may represent potential novel targets in migrating glioma cells having a stem cell phenotype.
